# Gene-set and proteomic signatures associated with survival after in-hospital cardiac arrest

**DOI:** 10.1016/j.resplu.2026.101359

**Published:** 2026-05-13

**Authors:** Jignesh K. Patel, Sam Parnia, Fumito Ichinose, Puja B. Parikh, Marc W. Halterman

**Affiliations:** a Department of Medicine, Stony Brook Renaissance School of Medicine, Stony Brook, NY, United States; b Department of Medicine, New York University Grossman School of Medicine, New York, NY, United States; c Department of Anesthesia, Critical Care and Pain Medicine, Massachusetts General Hospital and Harvard Medical School, Boston, MA, United States; d Jacobs School of Medicine and Biomedical Sciences, State University of New York at Buffalo, Buffalo, NY, United States

**Keywords:** Proteomics, Gene expression, Gene set enrichment analysis, Inflammation, Cardiac arrest, Survival, Mortality, Outcomes, Prognostication

## Abstract

**Background:**

In‑hospital cardiac arrest (IHCA) is associated with high mortality despite advances in resuscitation and post-cardiac arrest care. While individual inflammatory and neurologic biomarkers have been studied, less is known about coordinated proteomic and pathway‑level responses associated with survival after IHCA.

**Methods:**

In this prospective observational study, adult patients resuscitated after IHCA were enrolled at a tertiary academic medical center. Plasma samples were obtained at baseline (T0), 6 h (T6), and 24 h (T24) following return of spontaneous circulation (ROSC), when clinically feasible. High‑dimensional proteomic profiling was performed using the Olink® Explore 1536 platform. Survival‑associated proteins were identified using linear mixed‑effects models, and gene set enrichment analysis (GSEA) was performed to identify biologically coherent pathways.

**Results:**

Ninety‑five patients were enrolled. Due to early mortality and clinical constraints, analyzable samples were obtained from overlapping patient subsets at T0 (*n* = 24), T6 (*n* = 21), and T24 (*n* = 49). Individual protein‑level differences were limited at baseline. The greatest divergence between survivors and non‑survivors occurred at 6 h, characterized by enrichment of immune‑metabolic, mitochondrial, and transcriptional pathways. At 24 h, pathway enrichment narrowed toward chemokine signaling, GPCR‑mediated responses, and oxidative stress. Baseline pathway signals were nominal and did not meet false discovery rate thresholds.

**Conclusions:**

Survival following IHCA is associated with dynamic, time‑dependent proteomic and pathway‑level signatures, with the most pronounced biological divergence occurring early after resuscitation. These findings support the concept of time‑sensitive molecular phenotyping in post-cardiac arrest care and provide a foundation for future translational studies.

## Introduction

In-hospital cardiac arrest (IHCA) affects approximately 1% of hospitalized adults and remains associated with survival-to-discharge rates below 25%, despite decades of improvements in resuscitation science and post-cardiac arrest management.^1^, ^2^, ^3^, ^4^ Recent guidelines emphasize high-quality cardiopulmonary resuscitation, early defibrillation, and structured post-resuscitation care; however, substantial heterogeneity in outcomes persists even among patients receiving guideline-concordant care.^3^, ^4^, ^5^ Post-cardiac arrest syndrome is characterized by global ischemia–reperfusion injury, systemic inflammation, immune dysregulation, mitochondrial dysfunction, and secondary organ injury.^6^, ^7^ Prior human studies have largely focused on individual biomarkers – such as interleukin-6 (IL-6), neuron-specific enolase, neurofilament light chain, and S-100B – with modest prognostic performance and significant sensitivity to timing and confounding by illness severity.^8^, ^9^, ^10^, ^11^, ^12^, ^13^, ^14^, ^15^, ^16^, ^17^ These limitations highlight the need for approaches that capture coordinated biological responses rather than isolated molecular signals.

High-dimensional proteomic profiling offers an opportunity to define systemic biological programs associated with survival following IHCA. Rather than emphasizing single proteins, pathway-level analyses may better reflect integrated immune, metabolic, and stress-response states that evolve dynamically after resuscitation. Accordingly, the goal of the current investigation was to identify time-dependent proteomic signatures and gene-set-level biological processes associated with survival after IHCA.

## Methods

### Study population

Adult patients (age ≥18 years) experiencing IHCA requiring cardiopulmonary resuscitation for more than 5 min were prospectively enrolled between March 2014 and May 2019 at an academic tertiary-care medical center. Exclusion criteria included out-of-hospital cardiac arrest, trauma-related arrest, hospitalization in cardiothoracic or surgical intensive care units, or the presence of a do-not-resuscitate order without attempted resuscitation. Resuscitation and post-cardiac arrest care were performed in accordance with contemporaneous Advanced Care Life Support (ACLS) guidelines and institutional protocols.

### Ethics and regulatory approval

This study was conducted in accordance with the Declaration of Helsinki and all relevant institutional and national regulations. The study protocol was approved by the Stony Brook Institutional Review Board in 2012. Waiver of consent was requested for patients who did not survive cardiac arrest. Informed consent was obtained from surviving patients or their legally authorized representative.

### Outcomes

The primary outcome was survival to hospital discharge. A secondary outcome was favorable neurologic status at discharge, defined as Glasgow Outcome Score (GOS) of 4–5, determined by trained physicians through structured medical record review.

### Blood collection and proteomic analysis

Blood samples were obtained at baseline (i.e. at the time of arrest), and at 6, 24, 72, and 96 h following ROSC when clinically feasible. Within 30 min, the blood was centrifuged for 10 min at 2500 RPM to separate plasma. The aliquots were stored in a −80 °C freezer until the time of analysis. Proteomic profiling was performed using the Olink® Explore 1536 platform (Olink Proteomics, Inc.), which quantifies 1536 proteins using proximity extension assay technology and reports normalized protein expression (NPX) values on a log2 scale.

### Data pre-processing and statistical analysis

Bivariate analyses were performed based upon survival status. Chi-squared test (or Fisher’s exact test, when applicable) was used to compare differences in categorical variables and student’s *t*-test was used for continuous variables. Baseline comparisons are presented descriptively and were not adjusted for multiple testing.

Principal component analysis was utilized to examine the full protein expression data for all samples to identify potential outliers. The identification of proteins that change significantly across survival status was identified using a Linear Mixed Effects (LME) model. Given the higher number of samples at T0, T6, and T24, modeling data has been restricted to these time points. From the survival and gender LME model, 431 assays were found to be significant (adjusted *p*-value < 0.05). For every assay, a global test of significance was performed for the main effect of survival, time, and the interaction between them. Results were then adjusted for multiple testing using the Benjamini-Hochberg false discovery rate (FDR) method. Assays showing significance in any effect were further analyzed in a post hoc analysis to determine which specific contrasts were statistically significant. Post hoc results were generated for all proteins to compare the estimates between models at T0, T6, and T24. Heatmaps and volcano plots were generated to visualize protein expression differences between survivors and non-survivors at three timepoints: baseline (T0), 6 h (T6), and 24 h (T24).

### Gene set enrichment analysis

Gene set enrichment analysis (GSEA), a computation technique that determines whether an a priori set of genes (such as those related to a specific pathway or biological concept) show statistically significant differences between two biological states (in this case survival status) was also performed. Curated gene sets and ontological gene sets from the Molecular Signatures Database (MsigDB) were used to perform GSEA for T0, T6, and T24. Normalized enrichment scores (NES) and FDR *q*-values were calculated using permutation-based testing. Pathway-level findings were interpreted using conventional FDR significance thresholds (*q* < 0.05) with higher *q* values considered exploratory. The top pathways for each analysis were visualized as a heatmap and a bar graph. Bar graphs of the normalized enrichment score for the top gene sets were used to compare enrichment scores across gene sets.

## Results

### Patient characteristics and sample availability

Ninety‑five patients were enrolled at the time of IHCA. Due to early in‑hospital mortality and clinical or logistical constraints, analyzable proteomic samples were available from overlapping but non‑identical patient subsets at each timepoint: 24 patients at T0, 21 patients at T6, and 49 patients at T24. Baseline clinical characteristics stratified by survival status are presented in Table 1. Survivors and non‑survivors differed across several clinical domains, consistent with known prognostic factors in IHCA. Of the 95 patients (65% men and 35% women), 24 (25.3%) survived to hospital discharge while 71 (74.7%) did not.

**Table 1:** Medical history and clinical outcomes.

	**Overall** **(*n* = 95)**	**Died** **(*n* = 71)**	**Survived** **(*n* = 24)**	***P* value**
Age (years)	66 ± 16	67 ± 15	63 ± 17	0.239
Male gender	62 (65.3%)	43 (60.6%)	19 (79.2%)	0.098
Coronary artery disease	41 (43.2%)	26 (36.6%)	15 (62.5%)	0.027
Prior myocardial infarction	17 (17.9%)	13 (18.6%)	4 (16.7%)	1.000
Prior percutaneous coronary intervention	22 (23.2%)	15 (21.1%)	7 (29.2%)	0.420
**Diabetes mellitus**				
Without end organ damage	21 (22.1%)	16 (22.5%)	5 (20.8%)	0.862
With end organ damage	19 (20.0%)	15 (21.1%)	4 (16.7%)	0.773
Congestive heart failure	45 (47.4%)	34 (47.9%)	11 (45.8%)	0.862
Peripheral artery disease	14 (14.7%)	13 (18.6%)	1 (4.2%)	0.107
Cerebrovascular disease	15 (15.8%)	12 (16.9%)	3 (12.5%)	0.753
Advanced chronic kidney disease	29 (30.5%)	24 (33.8%)	5 (20.8%)	0.233
Chronic lung disease	32 (33.7%)	23 (32.4%)	9 (37.5%)	0.647
Charlson comorbidity score	5.8 ± 2.8	6.1 ± 2.7	4.9 ± 2.9	0.071
Initial rhythm				0.537
PEA/asystole	80 (84.2%)	61 (85.9%)	19 (79.2%)	
VT/VF	14 (14.7%)	9 (12.7%)	5 (20.8%)	
Other	1 (1.1%)	1 (1.4%)	0 (0.0%)	
Defibrillation	40 (42.1%)	30 (42.3%)	10 (43.5%)	0.918
CPR duration (min)	18.7 ± 15.4	20.8 ± 16.7	12.4 ± 7.6	0.022

### Differential proteomic signatures by survival status

**Fig. 1:**
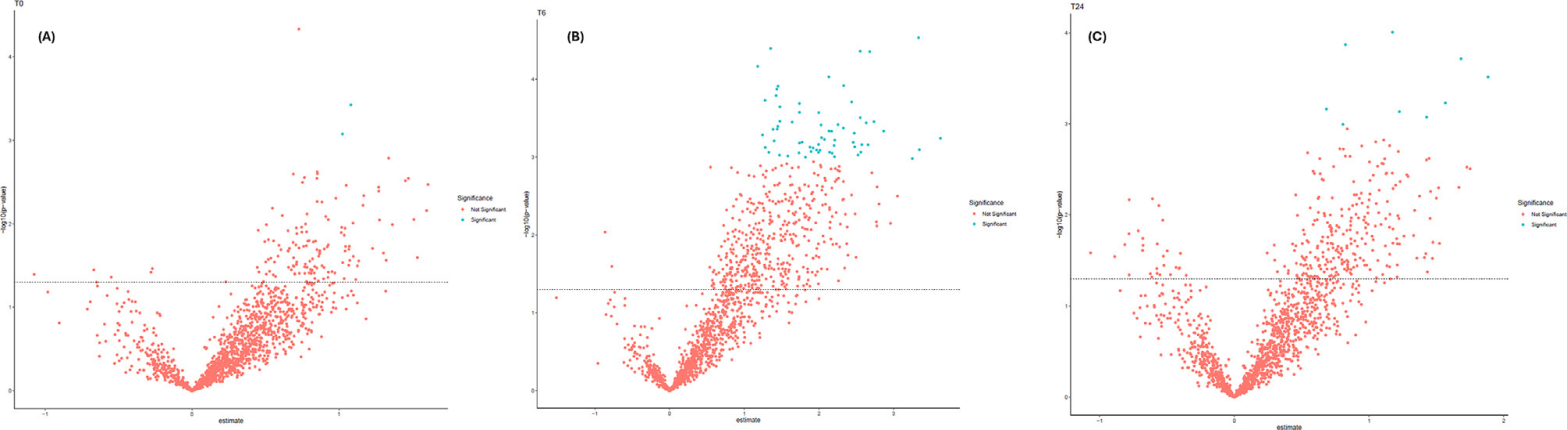
**Survival‑associated proteomic signatures over time**. Volcano plots illustrate time‑dependent differences in protein expression between survivors and non‑survivors following in‑hospital cardiac arrest. Minimal separation at baseline (T0) contrasts with marked divergence at 6 h (T6), indicating early activation of survival‑associated immune, metabolic, and stress‑response processes. By 24 h (T24), the magnitude and number of differentially expressed proteins decrease, suggesting consolidation of a narrower late‑phase molecular signature.

**Fig. 2:**
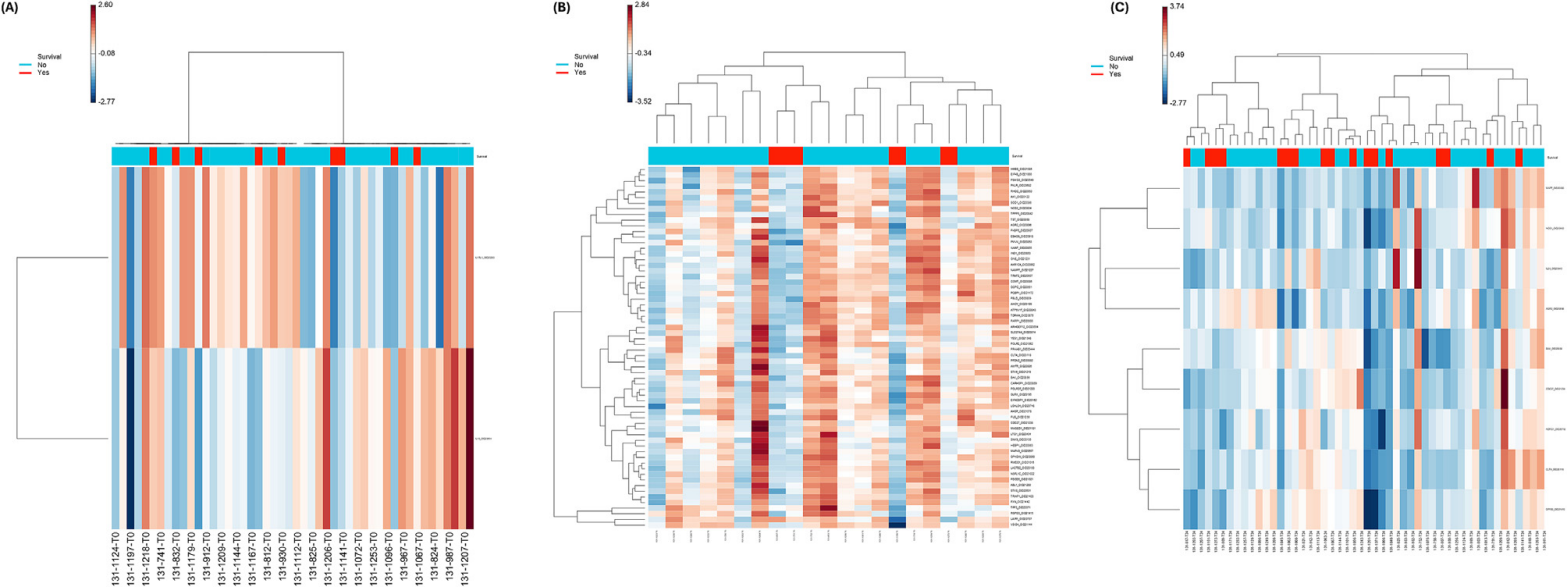
**Temporal evolution of outcome‑associated proteomic patterns**. Heatmaps demonstrate distinct and evolving proteomic patterns associated with survival following in‑hospital cardiac arrest. The strongest separation between survivors and non‑survivors occurs at 6 h, consistent with widespread immune‑metabolic and mitochondrial perturbations during early post‑resuscitation injury. At 24 h, a smaller but coherent protein signature persists, reflecting later inflammatory and cellular stress responses.

Heatmaps and volcano plots were generated to visualize protein expression differences between survivors and non-survivors at three timepoints: baseline (T0), 6 h (T6), and 24 h (T24) (Figs. 1A–C and 2A–C). At baseline (T0), individual protein‑level differences between survivors and non‑survivors were minimal. Volcano plot (Fig. 1A) demonstrated limited separation, and only a small number of proteins met adjusted statistical thresholds.

In contrast, at 6 h following ROSC, proteomic divergence between outcome groups was most pronounced. Numerous proteins related to immune regulation, cellular metabolism, mitochondrial function, and stress responses differed by survival status, reflected by broad separation on the volcano plot (Fig. 1B) and coherent clustering patterns on the heatmap (Fig. 2B). These findings indicate a peak in immune-metabolic reprogramming and apoptotic signaling at this timepoint.

By 24 h, the number of differentially expressed proteins decreased, with a more focused set of proteins associated with inflammation, oxidative stress, and cellular trafficking. Several proteins persisted across both T6 and T24, suggesting a stable core molecular signature extending beyond the early response phase. Together, these data highlight T6 as the most informative timepoint for outcome discrimination, with T24 capturing a more refined late-phase signature.

**Fig. 3:**
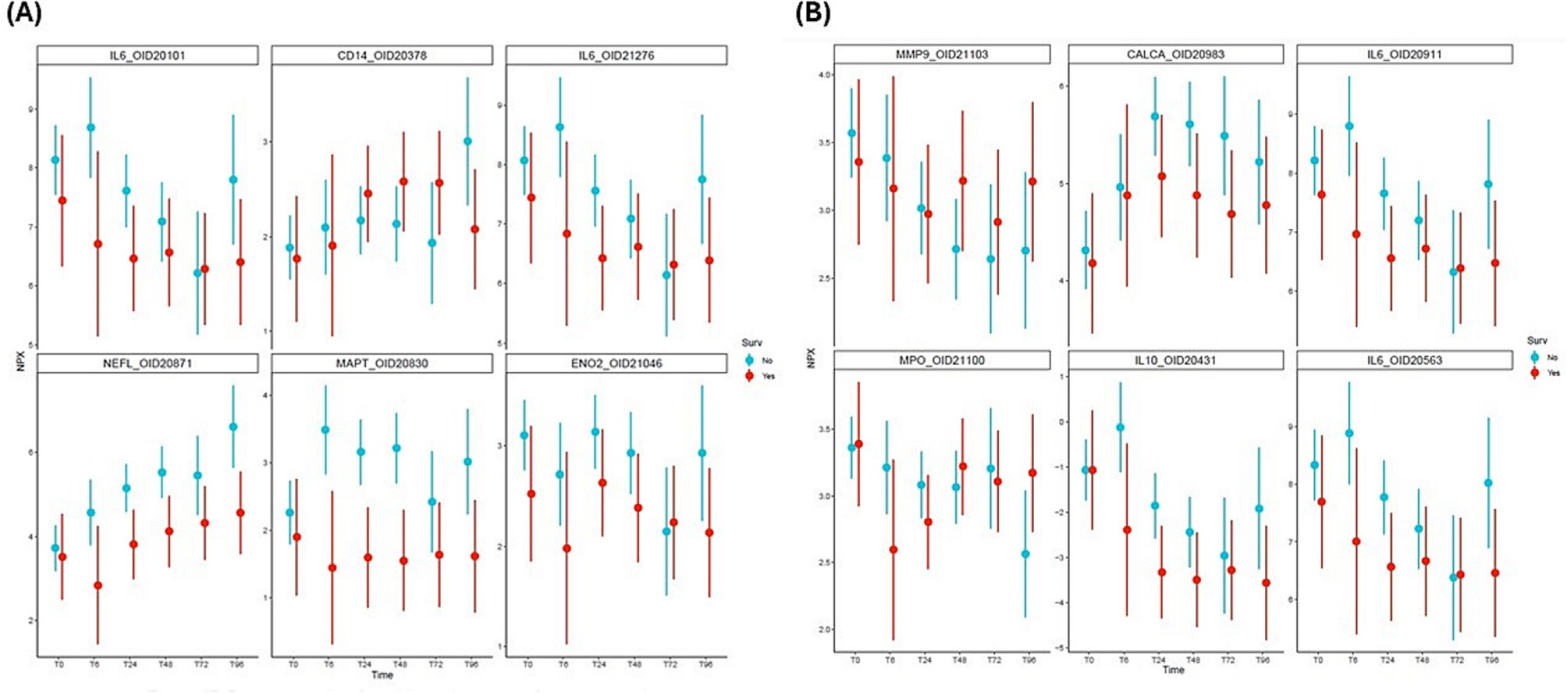
**Established biomarkers in the context of global proteomic responses**. Point‑range plots show temporal expression patterns of selected inflammatory and neurologic injury biomarkers commonly studied after cardiac arrest. These markers demonstrate variability across timepoints and outcome groups but capture only a subset of the broader molecular programs identified through high‑dimensional proteomic profiling. Statistical inference for survival‑associated differences was performed using linear mixed‑effects models and is reported in the main analyses.

Additional point-range plots were generated for known biomarkers of interest, including IL6 (Fig. 3A, B) to show temporal expression patterns of selected inflammatory and neurologic injury biomarkers commonly studied after cardiac arrest. These plots are intended to contextualize established biomarkers within the broader proteomic response rather than to provide independent hypothesis testing.

### Gene set enrichment analysis

**Fig. 4:**
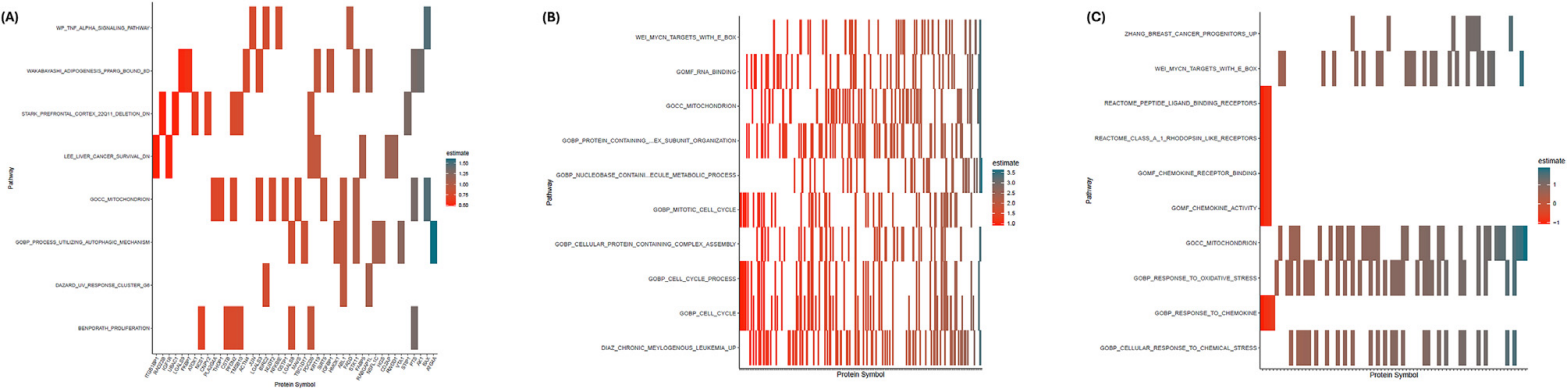
**Pathway‑level biological themes distinguishing survivors and non‑survivors**. Heatmaps of top gene sets from gene set enrichment analysis illustrate coordinated biological programs associated with survival across timepoints. Early timepoints highlight stress‑response and mitochondrial‑associated pathways, while the 6‑h timepoint shows peak enrichment of immune‑metabolic and transcriptional programs. At 24 h, enrichment emphasizes chemokine signaling, GPCR‑mediated responses, and oxidative stress, reflecting a transition to later inflammatory and immune‑trafficking processes.

**Fig. 5:**
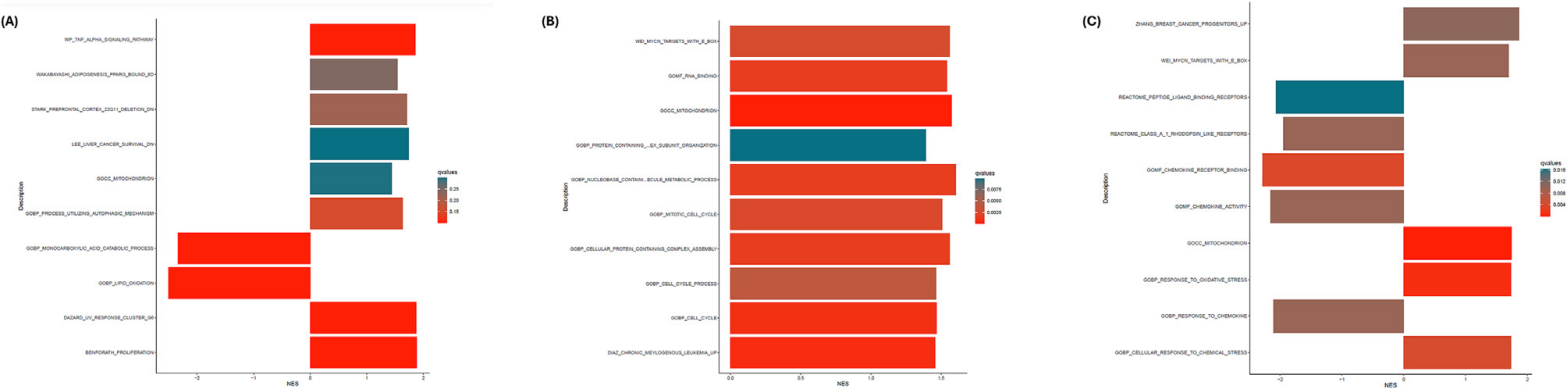
**Dynamic shift in enriched biological pathways after resuscitation**. Bar plots of normalized enrichment scores summarize the temporal evolution of survival‑associated pathways following in‑hospital cardiac arrest. Mitochondrial, metabolic, and cell‑cycle–related pathways predominate early after resuscitation, whereas chemokine‑mediated and GPCR‑linked signaling pathways become more prominent at later timepoints, highlighting the dynamic nature of post-cardiac arrest biology.

Table 2 depicts the top-ranked gene sets distinguishing survivors from non-survivors at T0, T6, and T24. At baseline (T0) several pathways related to cellular stress, apoptosis, mitochondrial localization, and TNF‑α signaling showed nominal enrichment; however, none met conventional FDR thresholds (*q* ≈ 0.15–0.25) and were therefore considered exploratory (Figs. 4A and 5A). At 6 h, GSEA revealed statistically significant enrichment of pathways related to mitochondrial function, RNA processing, cellular metabolism, and cell cycle regulation, representing the most robust pathway‑level divergence between survivors and non‑survivors (Figs. 4B and 5B). At 24 h, pathway enrichment shifted toward chemokine signaling, GPCR‑mediated responses, oxidative stress, and immune trafficking. Mitochondrial‑associated pathways remained prominent across timepoints, underscoring their central role in post‑arrest biology (Figs. 4C and 5C). A summary of known functions of the listed genes, particularly in the context of cardiac arrest or cardiovascular disease, has been provided in Table 3.

**Table 2:** Top 10 gene sets from gene set enrichment analysis at each time point.

**Description**	**NES**	***p* value**	***q* value**
***0 HOURS***			
WP TNF ALPHA SIGNALING PATHWAY	1.855184	0.0000235	0.1012339
BENPORATH PROLIFERATION	1.883912	0.0000483	0.1012339
GOBP LIPID OXIDATION	−2.501907	0.0000965	0.1012339
DAZARD UV RESPONSE CLUSTER G6	1.872036	0.0001010	0.1012339
GOBP MONOCARBOXYLIC ACID CATABOLIC PROCESS	−2.336587	0.0001033	0.1012339
GOBP PROCESS UTILIZING AUTOPHAGIC MECHANISM	1.628349	0.0001831	0.1495663
STARK PREFRONTAL CORTEX 22Q11 DELETION DN	1.706157	0.0003039	0.2128533
WAKABAYASHI ADIPOGENESIS PPARG BOUND 8D	1.536909	0.0003942	0.2415354
GOCC MITOCHONDRION	1.438295	0.0005450	0.2968255
LEE LIVER CANCER SURVIVAL DN	1.742488	0.0006115	0.2997760

***6 HOURS***			
GOCC MITOCHONDRION	1.574168	<0.0000001	0.0000560
DIAZ CHRONIC MEYLOGENOUS LEUKEMIA UP	1.456638	0.0000002	0.0005047
GOBC CELL CYCLE	1.466733	0.0000004	0.0006505
GOMF RNA BINDING	1.540571	0.0000014	0.0013904
GOBP CELLULAR PROTEIN CONTAINING COMPLEX ASSEMBLY	1.559450	0.0000015	0.0013904
GOBP NUCLEOBASE CONTAINING SMALL MOLECULE METABOLIC PROCESS	1.602555	0.0000019	0.0014180
GOBP MITOTIC CELL CYCLE	1.506927	0.0000035	0.0022413
WEI MYCN TARGETS WITH E BOX	1.560615	0.0000044	0.0024906
GOBP CELL CYCLE PROCESS	1.462110	0.0000077	0.0038890
GOBP PROTEIN CONTAINING COMPLEX SUBUNIT ORGANIZATION	1.388703	0.0000216	0.0098232

***24 HOURS***			
GOCC MITOCHONDRION	1.739559	<0.0000001	0.0000366
GOBP RESPONSE TO OXIDATIVE STRESS	1.732470	0.0000003	0.0008058
GOMF CHEMOKINE RECEPTOR BINDING	−2.284606	0.0000023	0.0034626
GOBP CELLULAR RESPONSE TO CHEMICAL STRESS	1.734983	0.0000036	0.0041127
GOBP RESPONSE TO CHEMOKINE	−2.108641	0.0000106	0.0097487
REACTOME CLASS A 1 RHODOPSIN LIKE RECEPTORS	−1.950209	0.0000139	0.0097487
GOMF CHEMOKINE ACTIVITY	−2.157966	0.0000169	0.0097487
WEI MYCN TARGETS WITH E BOX	1.691830	0.0000169	0.0097487
ZHANG BREAST CANCER PROGENITORS UP	1.864238	0.0000205	0.0105076
REACTOME PEPTIDE LIGAND BINDING RECEPTORS	−2.067340	0.0000380	0.0162304

**Table 3:** Summary of function of identified genes involved in cardiac arrest.

**MAPT (Microtubule-Associated Protein Tau)**
• MAPT encodes the protein Tau, which stabilizes microtubules. • Abnormal phosphorylation of Tau can lead to neurodegenerative diseases like Alzheimer's. • Microtubule stability and function could be affected during ischemia–reperfusion injury, which might impact cardiac cell survival. • Tau serves as a promising biomarker for assessing brain injury after cardiac arrest, with potential utility in prognosis and guiding treatment.

**NOS1 (Nitric Oxide Synthase)**
• NOS1 produces nitric oxide (NO) that plays a significant role in vascular tone, blood flow regulation, and protecting against ischemia–reperfusion injury. • In the context of cardiac arrest, NO can help mitigate tissue damage by reducing oxidative stress and improving blood flow.

**IL24 (Interleukin 24)**
• IL24 implicated in processes like cell survival, apoptosis, and inflammation. • IL24 may act within the broader cytokine network to either amplify or dampen inflammation, depending on the context. • Its role could be to help balance the inflammatory response, potentially reducing secondary damage caused by excessive inflammation.

**AGR2 (Anterior Gradient 2)**
• AGR2 is a protein disulfide isomerase involved in protein folding and cellular stress responses. • It is also implicated in cell survival, particularly under stressful conditions. • AGR2 might play a role in protecting cardiac cells during ischemic stress, though its specific role in cardiac arrest is not well-documented.

**BAX (BCL2-Associated X Protein)**
• BAX is a pro-apoptotic protein that promotes cell death by facilitating the release of cytochrome *c* from mitochondria. • During cardiac arrest, ischemia–reperfusion injury can lead to increased BAX activity, resulting in apoptosis of cardiac cells and contributing to tissue damage.

**CDC27 (Cell Division Cycle 27)**
• CDC27 is part of the anaphase-promoting complex (APC), which regulates cell cycle progression. • While its direct role in cardiac arrest is not well-defined, disruptions in cell cycle regulation could contribute to cellular responses following ischemia–reperfusion injury.

**NDRG1 (N-myc Downstream-Regulated Gene 1)**
• NDRG1 is involved in stress responses, including hypoxia and cellular differentiation. • It plays a protective role under stress conditions like ischemia, making it potentially important in the context of cardiac arrest, where hypoxic conditions prevail.

**CLTA (Clathrin Light Chain A)**
• CLTA is involved in clathrin-mediated endocytosis, a process crucial for membrane recycling and cellular signaling. • During cardiac arrest, disruptions in cellular signaling and membrane dynamics can affect cardiac function, where proteins like CLTA might play a role.

**DPY30 (Dpy-30 Homolog)**
• DPY30 is part of the COMPASS complex, which is involved in histone methylation and gene expression regulation. • While its role in cardiac arrest is not well characterized, epigenetic regulation is increasingly recognized as important in the response to ischemic injury and could influence the outcomes of cardiac arrest.

## Discussion

In this time-resolved proteomic study of in-hospital cardiac arrest, survival was associated with dynamic, pathway-level molecular responses that evolved over the first 24 h following resuscitation. At baseline (T0), only a limited set of proteins differentiated survivors from non-survivors, suggesting that pre-arrest or immediate post-arrest inflammatory tone may carry modest prognostic value. However, pathway analysis at T0 suggested nominal enrichment of stress-, apoptotic-, and autophagy-related programs, which did not meet statistical significance after multiple-testing correction and should be interpreted as exploratory signals.

A key finding of this study is the marked biological divergence observed at 6 h after resuscitation. This timepoint demonstrated the strongest enrichment of immune‑metabolic and mitochondrial pathways, suggesting that early cellular adaptation to ischemia‑reperfusion injury may play a critical role in recovery. The prominence of mitochondrial and immune-metabolic pathways at 6 h aligns with the known timeline of ischemia-reperfusion injury and secondary inflammatory amplification following cardiac arrest. Importantly, this period precedes routine neurologic prognostication and overlaps with early post‑arrest decision‑making, highlighting its potential translational relevance.

Compared with established post-cardiac arrest biomarkers—such as IL‑6, NSE, and neurofilament light^8^, ^9^, ^10^, ^11^, ^12^, ^13^, ^14^, ^15^, ^16^—these proteomic signatures capture broader systems‑level responses rather than single mechanistic axes. This may help explain why single‑biomarker strategies have demonstrated limited prognostic discrimination and supports further investigation into molecular phenotyping approaches in critical illness.

These findings suggest that early post-arrest molecular responses—particularly at 6 h—may offer prognostic insight and identify mechanistic targets for intervention.

Our findings parallel and extend prior multi-omics studies that have applied volcano plots, heat-map clustering, and high-dimensional analytical workflows to cardiac arrest populations. Stefanizzi et al.^18^ demonstrated that circular RNA expression patterns could clearly differentiate favorable from poor neurological outcome, with volcano plots identifying differentially expressed circRNAs and heat-map clustering confirming distinct transcriptomic signatures—an approach analogous to our proteomic clustering findings in IHCA. Beske et al.^19^ used volcano plots to characterize metabolomic shifts following IL-6 blockade in post-cardiac arrest patients, identifying inflammation-related metabolic pathways; similarly, our study highlights inflammation, immune signaling, and mitochondrial dysfunction as differentiating features between survivors and non-survivors. Eun et al.^20^ used transcriptomic heat-map clustering to separate cardiac-arrest survivors from non-survivors, with many immune-activation and neural-injury pathways overlapping proteins found in our analysis. Caudal et al.^21^ further demonstrated ischemia-driven transcriptomic alterations in myocardial tissue during sudden cardiac death, identifying mitochondrial and oxidative-stress pathways concordant with our proteomic observations. Finally, Nakashima et al.^22^ showed that machine-learning models using feature-importance heat-maps could predict out-of-hospital cardiac arrest, reinforcing the broader applicability of high-dimensional pattern-recognition methods—such as the volcano plots and heat-map analyses used in our study—for critical-illness prognostication. Collectively, these prior studies support the concept that integrative omics-based visualization methods reveal biologically meaningful mortality-associated signatures across transcriptomic, metabolomic, and proteomic layers.

This study has important limitations. Biospecimen availability varied across timepoints due to early mortality and clinical constraints, resulting in an unbalanced longitudinal design and survivor‑enriched later samples. The single‑center nature of the study may limit generalizability to other institutions with differing patient populations or care practices. Analyses were exploratory and unadjusted for clinical covariates, and findings should be interpreted as hypothesis‑generating rather than causal. This study lacked follow-up data and outcomes are limited to the in-hospital phase of care. Finally, baseline (T0) pathway signals should be interpreted cautiously. Although nominal trends suggested early stress‑ and apoptosis‑related processes, these findings did not meet statistical significance after multiple‑testing correction and should be viewed as exploratory. In contrast, pathway enrichment at later timepoints demonstrated statistically robust and biologically coherent separation.

Despite these limitations, our findings parallel prior omics-based cardiac arrest studies, including transcriptomic, metabolomic, and machine-learning biomarker analyses that used volcano plots and heatmap clustering to distinguish survivors from non-survivors^18^, ^19^, ^20^, ^21^, ^22^. Similar to these investigations, we identified coordinated pathway-level differences involving inflammatory signaling, mitochondrial function, and cellular stress responses.

## Conclusions

Survival following in‑hospital cardiac arrest is associated with dynamic, time‑dependent proteomic and pathway‑level biological signatures, with the greatest divergence occurring early after resuscitation. These findings provide a foundation for future multi‑center validation studies and support the potential role of time‑sensitive molecular phenotyping in advancing prognostication and therapeutic discovery in post-cardiac arrest care.

## Financial support

Feldstein Medical Foundation Grant; Stony Brook University Targeted Research Opportunity Grant; Stony Brook Department of Medicine Pilot Project Grant.

## CRediT authorship contribution statement

**Jignesh K. Patel:** Writing – review & editing, Writing – original draft, Visualization, Validation, Supervision, Software, Resources, Project administration, Methodology, Investigation, Funding acquisition, Formal analysis, Data curation, Conceptualization. **Sam Parnia:** Writing – review & editing. **Fumito Ichinose:** Writing – review & editing. **Puja B. Parikh:** Formal analysis. **Marc W. Halterman:** Writing – review & editing, Writing – original draft.

## Declaration of competing interest

The authors declare that they have no known competing financial interests or personal relationships that could have appeared to influence the work reported in this paper.
